# The Effects of Hysterectomy on Urinary and Sexual Functions of Women with Cervical Cancer: A Systematic Review

**DOI:** 10.1055/s-0042-1748972

**Published:** 2022-09-08

**Authors:** Mariana Alves Firmeza, Camila Teixeira Moreira Vasconcelos, José Ananias Vasconcelos Neto, Luiz Gustavo de Oliveira Brito, Flávio Mendes Alves, Natália Maria de Vasconcelos Oliveira

**Affiliations:** 1 Nursing Department, Universidade Federal do Ceará, Fortaleza, Ceará, Brasil; 2 Woman's Health Department, Universidade Federal do Ceará, Fortaleza, Ceará, Brasil; 3 Gynecology Department, Universidade Estadual de Campinas, Campinas, SP, Brasil

**Keywords:** uterine cervical neoplasms, hysterectomy, lower urinary tract symptoms, neoplasias do colo do útero, histerectomia, sintomas do trato urinário inferior

## Abstract

**Objective** This systematic review aims at describing the prevalence of urinary and sexual symptoms among women who underwent a hysterectomy for cervical cancer.

**Methods** A systematic search in six electronic databases was performed, in September 2019, by two researchers. The text search was limited to the investigation of prevalence or occurrence of lower urinary tract symptoms (LUTS) and sexual dysfunctions in women who underwent a hysterectomy for cervical cancer. For search strategies, specific combinations of terms were used.

**Results** A total of 8 studies, published between 2010 and 2018, were included in the sample. The average age of the participants ranged from 40 to 56 years, and the dysfunctions predominantly investigated in the articles were urinary symptoms ( *n* = 8). The rates of urinary incontinence due to radical abdominal hysterectomy ranged from 7 to 31%. The same dysfunction related to laparoscopic radical hysterectomy varied from 25 to 35% and to laparoscopic nerve sparing radical hysterectomy varied from 25 to 47%. Nocturia ranged from 13%, before treatment, to 30%, after radical hysterectomy. The prevalence rates of dyspareunia related to laparoscopic radical hysterectomy and laparoscopic nerve sparing radical hysterectomy ranged from 5 to 16% and 7 to 19% respectively. The difficulty in having orgasm was related to laparoscopic radical hysterectomy (10 to 14%) and laparoscopic nerve sparing radical hysterectomy (9 to 19%).

**Conclusion** Urinary and sexual dysfunctions after radical hysterectomy to treat cervical cancer are frequent events. The main reported disorders were urinary incontinence and dyspareunia.

## Resumo

**Objetivo** Essa revisão sistemática visa descrever a prevalência de sintomas urinários e sexuais entre mulheres submetidas à histerectomia por câncer cervical.

**Métodos** Uma pesquisa sistemática foi realizada em seis bases de dados eletrônicas, em setembro de 2019, por dois pesquisadores. A busca foi limitada à investigação da prevalência e ocorrência de sintomas do trato urinário baixo e disfunções sexuais em mulheres após histerectomia por câncer cervical. Como estratégia de busca foi utilizada uma combinação específica de termos apenas em inglês.

**Resultados** Um total de 8 estudos, publicados entre 2010 e 2018, foram incluídos na amostra. A idade média dos participantes foi de 40 a 56 anos, e as principais disfunções investigadas pelos artigos foram sintomas urinários ( *n* = 8). Na literatura analisada, as taxas de incontinência urinária ligadas à histerectomia abdominal radical variaram de 7 a 31%. A mesma disfunção, para histerectomia radical laparoscópica, variou de 25 a 35%, e de 25 a 47% para histerectomia radical laparoscópica poupadora de nervo. A taxa de noctúria variou de 13%, antes do tratamento, a 30%, após histerectomia radical. A prevalência de dispareunia associada à histerectomia radical laparoscópica foi de 5 a 16%. Já a taxa de dispareunia relatada pós-histerectomia radical laparoscópica poupadora situou-se entre 7 e 19%. A dificuldade de alcançar o orgasmo foi relatada após histerectomia radical laparoscópica, variando de 10 a 14%, e também na histerectomia radical laparoscópica poupadora de nervo, variando de 9 a 19%.

**Conclusão** Disfunções urinárias e sexuais após histerectomia para tratamento do câncer cervical são eventos frequentes. As principais desordens relatadas foram incontinência urinária e dispareunia.

## Introduction

Cervical cancer is the fourth most common female cancer worldwide and the second in low/middle-income countries (LMICs). 1 In Brazil, it is the third most frequent and the fourth leading cause of death for women. 2 The treatment of cervical cancer begins with surgery or radiation therapy, with or without chemotherapy. Surgery is indicated in the early stages. Surgical technique is selected according to disease staging: cervical conization, total simple hysterectomy, or radical hysterectomy. 3 Among radical hysterectomies, the most common is the type III technique, involving more extensive removal of the upper vagina, as well as uterosacral ligaments and bilateral parametrium. 4

Although there is a high survival rate for treated, early-stage, node-negative cervical cancer, radical hysterectomy leads to a major morbidity, in particular, regarding the urinary and sexual functions. 5 6 7 8 In addition, despite the early transient changes in pelvic organ functions, after radical hysterectomy, the long-term prevalence of symptoms and the extent of morbidity associated with the procedure have not been well established. 9 Selcuk et al. 9 affirm that hysterectomy impacts on the quality of life (QoL), regarding aspects of pelvic floor functions, especially in women submitted to radical hysterectomy. Urinary symptoms (retention, urgency) and sexual dysfunctions are as uncomfortable as challenging to overwhelmed patients. 9

Surgical treatment of women with cervical cancer causes significant injury to the pelvic floor. In fact, hysterectomy impairs the anatomical relationship between the pelvic organs (bladder, uterus, bowel, and vagina), supportive structures, and local nerve supply, which disrupts the pelvic floor normal function. 5 It is thought that surgical damage to the pelvic autonomic nerves plays a critical role in urinary and sexual dysfunctions. 5 8 10 11

Differences on the surgical procedures will involve varying degrees of dissection and disruption of the neuroanatomy of the pelvic organs. Such variations are important to be pointed out, for they may be related to the studied outcomes. Thus, we aimed at this systematic review to describe the prevalence of urinary and sexual symptoms among women who underwent a hysterectomy for cervical cancer.

## Methods

This study was undertaken in accordance with the Preferred Reporting Items for Systematic Reviews and Meta-Analysis (PRISMA) checklist. 12

A systematic search of the MEDLINE/Pubmed, SCIELO, LILACS, CINAHL, SCOPUS, and WEB OF SCIENCE databases was performed from September 1 to 7, 2019 by two researchers (M. A. F. and L. G. B. M. M.) to retrieve all the manuscripts that contained information on the prevalence of urinary disorders and sexual symptoms in women after cervical cancer hysterectomy. A manual search of reference lists was also performed. For search strategies, the following combinations of terms were used: *hysterectomy* OR *hysterectomy, vaginal* AND *uterine cervical neoplasms* OR *cervical cancer* OR *cervical neoplams* OR *genital neoplams, female* AND *pelvic floor disorders* OR *pelvic floor* , *hysterectomy* OR *hysterectomy, vaginal* AND *pelvic floor disorders* OR *pelvic floor* , *hysterectomy* OR *hysterectomy, vaginal* AND *uterine cervical neoplams* OR *cervical cancer* OR *cervical neoplasms* OR *genital neoplams* , female AND *diurnal* *enuresis* OR *nocturnal enuresis* OR *urine* AND *sexual dysfunction* OR *dyspareunia* OR *sexual* *behavior* OR *libido* OR *orgasm* / *hysterectomy* OR *hysterectomy* , *vaginal* AND *diurnal enuresis* OR *nocturnal* *enuresis* OR *urine* AND *sexual dysfunction* OR *dyspareunia* OR *sexual behavior* OR *libido* OR *orgasm* .

The search was limited to the investigation of prevalence or occurrence of LUTS and sexual dysfunctions in women who underwent a hysterectomy for cervical cancer. There was no limit regarding the publication period. Articles were excluded if they were duplicates, reviews, case studies, or commentaries.

We included randomized controlled trials (RCTs) and observational studies. The PICO criteria determined all parameters: women, aged between 18 and 65 years, who underwent hysterectomy with pelvic and/or paraaortic lymphadenectomy for cervical cancer (IA2, IB1, IB2, IIA, IIB), with or without radiotherapy (RT) or chemotherapy (QT), and who indicated urinary and sexual symptoms. Studies comparing surgical techniques for treating cervical cancer for assessing the onset of urinary or sexual dysfunctions were also included.

**Fig. 1:**
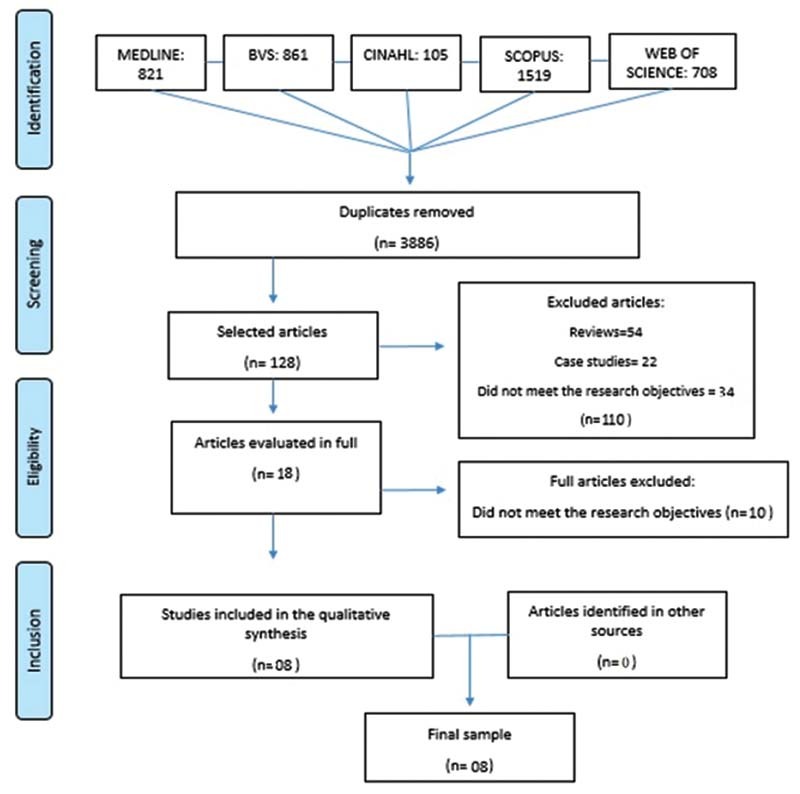
Flow referring to the selection process of the systematic review studies, adapted from PRISMA-ScR.

In the initial research, 4,015 studies were identified, out of which 3,886 duplicates were excluded. One hundred and ten out of 128 articles screened for eligibility were excluded, after the reading of the abstract, as well as 54 reviews, 22 case-studies, and others 34 papers, which did not meet the research goal. Twenty-seven articles were potentially eligible for inclusion in this review and, therefore, were read in full. After reading and analyzing them, 19 studies were excluded, resulting in 8 articles included in this review ( Fig. 1 ).

The search was conducted from the 1st to 7 ^th^ of September 2019 by two authors (M. A. F. and L. G. B. M. M.). The JBI Critical Appraisal Checklist for Studies Reporting Prevalence Data was used by the former author for assessment of risk of bias of the selected studies (criteria and appraisals are provided in supporting information) ( Charts 1 and 2 ). According to this instrument, each question presents four options: yes (Y), no (N), unclear (U), and not applicable (N/A). The calculation of the percentage of risk of bias was established out of the amount of Y that was selected in the checklist. When the N/A response was selected, the question was not considered in the calculation, according to the guidelines of the Joanna Briggs Institute. 13 Up to 49% of the calculation was considered a high risk of bias. From 50 up to 70%, the risk was considered moderate, and above 70%, the risk of bias was considered low. 13

The critical evaluation was discussed with the other authors who agreed with the study appraisals. A standardized data extraction sheet was developed and filled in, to extract data concerning the study design, population and sample, results, outcomes, and conclusions.

Symptoms of urinary and sexual dysfunction were those described by the *International Urogynecological Association (IUGA)/International Continence Society (ICS)* . 14 Data on urinary and sexual symptoms researched in the articles should be recorded and reported as: (1) the subject's observations (symptoms); (2) quantification of symptoms; (3) clinician's observations (anatomical and functional); (4) quality of life; and (5) socioeconomic measures. 15

Primary outcomes: prevalence of urinary symptoms (lower urinary tract symptoms, urinary incontinence, stress urinary incontinence, urge urinary incontinence, nocturia, and urinary tract infections) before and after hysterectomy. Prevalence of sexual symptoms (sexual dysfunction, dyspareunia, difficulty in having orgasm) before and after hysterectomy. 14

Secondary variables: age, type of surgery, instruments used to measure symptoms and period in which symptoms were measured.

Finally, data were categorized and organized in tables and figures, according to prevalence, associated factors, and impact on quality of life. A qualitative synthesis of all the studies was performed and included in the final sample, describing the study results organized by study characteristics and quality appraisal, prevalence, risk factors, and impact on urinary and sexual symptoms and on quality of life. After these evaluations, the selected studies were submitted to a statistical analysis to verify the possibility of constructing a meta-analysis, which would increase the accuracy and the evidential power of the results. However, this step was not possible due to heterogeneity of the methods, study samples, questionnaires, definition of symptoms, and reporting of results.

## Results

### Study Characteristics and Quality Appraisal

According to the search strategy adopted, 4,015 studies were identified, 3,886 of which were duplicates and, therefore, excluded. Title and abstracts of the 128 remaining papers were assessed to determine whether the study was adequate to be included, resulting in 110 articles excluded. Thus, 27 articles were fully evaluated for eligibility and 19 studies were excluded according to the eligibility criteria.

A total of 8 studies, published between 2010 and 2018, were included in the sample. The average age of the participants ranged from 40 to 56 years and the dysfunctions predominantly investigated by the articles were urinary symptoms ( *n* = 8). Five studies reported, in addition to data related to voiding dysfunction, female sexual dysfunction. 9 16 17 18 19 20 21 22 23 24 25 26 27 28 29 30 31 32 33 34 35 36 37 38 Only five studies used validated questionnaires in their data collections, the rest used standardized instruments in their respective collection fields ( Charts 1 and 2 ). The studies had a variation in patient follow-up from 3 months to 9 years. Most studies were performed in Europe. 16 27 38 39

Four retrospective cohort studies have reported oncological results and complications from various treatment modalities for various stages of cervical cancer. 9 27 36 37 Prospective cohorts, on the other hand, assessed the influence of hysterectomy on the pelvic floor regarding morbidity secondary to urinary and sexual symptoms. 16 22 38 39

The cross-sectional study that comprised the sample in this review was part of an original study and used questionnaires to assess urinary and sexual symptoms in women undergoing hysterectomy, with a follow-up of up to 9 years. 16

Charts 1 and 2 list publications that show the prevalence of urinary and sexual dysfunctions posthysterectomy. It is noteworthy that there are methodological differences between the studies regarding the following aspects: study designs, reason for the hysterectomy (type of cancer), surgical route (abdominal, laparoscopic, vaginal, etc.), age of the women, time of surgery, and questionnaires used and follow-up period.

### Prevalence of Urinary Symptoms

The rates of urinary incontinence due to radical abdominal hysterectomy ranged from 7 to 31%. The same dysfunction related to laparoscopic radical hysterectomy varied from 25 to 35%, and from 25 to 47% when related to laparoscopic nerve sparing radical hysterectomy, varied. Finally, relating stress urinary incontinence to radical abdominal hysterectomy, rates vary from 7 to 11%. Supracervical hysterectomy, in turn, when related to the rates of appearance of stress incontinence, had no variation (11%), urge incontinence ranged from 7, before surgery, and 45%, after surgery, in radical abdominal hysterectomy, and 5% after supracervical hysterectomy. Nocturia ranged from 13, before treatment, to 30% after the radical hysterectomy ( Charts 1 and 2 ).

### Prevalence of Sexual Symptoms

The prevalence rates of dyspareunia related to laparoscopic radical hysterectomy and laparoscopic nerve sparing radical hysterectomy ranged from 5 to 16% and 7 to 19% respectively. The difficulty in having orgasm was related to laparoscopic radical hysterectomy (10 to 14%) and laparoscopic nerve sparing radical hysterectomy (9 to 19%) ( Charts 1 and 2 , supplementary material).

### Evaluation of Studies

All 8 articles were analyzed, according to the type of study, through the JBI critical appraisal checklist, which aims at assessing the methodological quality of a study and determining the extent to which the authors addressed the possibility of bias in its design, conduct and analysis. Within the sample of this review, seven articles had a low risk of bias, and one article showed a moderate risk of bias ( Charts 1 and 2 , supplementary material).

## Discussion

This systematic review was designed to assess the prevalence of urinary and sexual symptoms after the different types of hysterectomy. It was agreed that the observations or symptoms of the patients should include evaluations with validated questionnaires; however, three of the selected studies used non validated questionnaires. There is a critical need for validated and reliable instruments for symptom assessment in all types of pelvic floor disorders, which may include standardized interviews, questionnaires, symptom diaries, and other techniques for collecting qualitative and quantitative data. 19

The importance of using validated questionnaires in scientific research consists of attending to what the researcher proposes to unveil and of having coherence in the methodological processes and consistency of the results, allowing to analyze the existence of a logic between the proposed instruments and the objectives of research. The use of validated instruments in health studies is imperative to verify the need for intervention in some process. It is necessary to guarantee two characteristics of the measuring instruments: validity and reliability. Ensuring the validity of the instrument means statistically proving that the questionnaire really measures what it proposes, and reliability can be defined as the reproducibility of that measure. 20

In addition, in three studies, patients already had urinary and/or sexual symptoms before the surgical procedure. 16 21 22 23 Epidemiological studies show that the prevalence of voiding complaints among women aged 40 to 56 years can vary between 7 and 47% ( Charts 1 and 2 , supplementary material), and the prevalence of sexual symptoms in the same age group varies between 5 and 19% ( Charts 1 and 2 , supplementary material). Pelvic floor dysfunctions usually occur due to multiparity, obesity, previous pelvic surgery (such as hysterectomy), and behavioral factors. These changes lead to dysfunctions such as urinary (UI) and fecal (IF) incontinence, pelvic organ prolapses (POPs), constipation, and sexual dysfunctions. 24

Hysterectomy remains the main treatment modality for early cervical cancer, due to the effects of radiation on ovarian function and vaginal mucosa integrity. Although the 5-year survival is greater than 90% for negative lymph node disease, the procedure causes significant morbidity for patients, including on the pelvic floor, such as, for example, urinary and sexual dysfunctions. 25 Many studies in this review pointed out that any type of hysterectomy can result in more pelvic floor symptoms. 9 21 26

Although the literature mentions hysterectomy as a risk factor for the development of urinary dysfunctions, these symptoms are frequently related to other factors, such as pregnancy, childbirth, and climacteric period. Chen et al. 39 reported bladder dysfunction in oncological patients related to pathological changes in the detrusor muscle and injuries to bladder autonomic innervation by vaginal, paravaginal, and parametrial resection. These changes can lead to increase of urinary frequency, urinary urgency, and decrease of bladder compliance, symptoms that could persist for 6 to 12 months after surgery.

A study that compared the prevalence of these disorders among women with cervical cancer who underwent surgery and radiotherapy found that there were urinary complications in both groups, but sexual disorders, in the radiotherapy group, were more exacerbated in young and obese patients, while surgery-related disorders were more prevalent in the elderly and obese. 27 This data corroborates with the literature, when it states that some clinical and demographic characteristics may be associated with increased intensity of bladder and sexual symptoms in patients with gynecological cancer, such as, obesity and a low level of education. 28

Cosson et al. (2001) 22 also stated that many long-term complications, which appear in the first 4 years after hysterectomy, cannot be attributed to the intervention. There are confounding factors, such as age and hormonal changes. 22 In the writing stages of this review, a shortage of studies about these factors was noted. They are relevant considering the sample profile and high climacteric symptoms prevalence. Therefore, we emphasize the need for more studies evaluating such variables.

When it comes to laparoscopic hysterectomies, we noticed that the variations in the rates of voiding dysfunction were much more significant than the rates of sexual problems presented by women. In addition, this type of surgery, compared with other surgical techniques, causes less morbidity to the female pelvic floor. 21

Sensory innervation lesion of the bladder, as well as damage to the autonomic pelvic plexus, can occur during hysterectomy, resulting in urinary dysfunction. Terminal bundles of the bilateral plexuses innervate the vagina and the proximal bladder and can be damaged during surgery, resulting in a defective closing mechanism of the proximal urethral sphincter. 29

The same authors also emphasize that radical hysterectomy for the treatment of cervical cancer causes significant morbidity to the pelvic floor, and this is related to the radicality of the parametrial and vaginal resection with partial denervation resulting from the pelvic viscera and it is not just explained by the removal of the uterus. 29 30 In addition, when surgery is associated with adjuvant radiotherapy, the prevalence of self-reported urinary and sexual symptoms increases. 29

The results obtained in this review also show that the treatment against early cervical cancer results in an increase of the sexual and urinary symptoms of the patients, regardless of the surgical procedure. 22

There may be consequences after hysterectomy, in the quality of the woman's sexual life, in her emotional conditions, and in the quality of the relationship established with her partner. The need to perform this surgery, in many cases, causes conflicting, traumatic, insecure emotions, and anxiety, generating important changes in sexual patterns and desire. 31

In Brazil, each year, many women receive an indication for hysterectomy. In 2017, 122 hysterectomies were performed per 100 thousand women over the age of 20 years. It is estimated that between 20 and 30% of women will undergo this procedure until the 6th decade of life. 32 These patients may present changes in self-image and depressive symptoms, due to conceptions about the uterus that are closely linked to the woman's sexuality.

In addition to the emotional aspects, anatomical changes also occur in the pelvis, which can lead to changes in the size and diameter of the vagina (leading to difficulty in vaginal penetration and dyspareunia) and reduction of circulating hormone levels (leading to decreased sexual desire, vaginal dryness, and lower frequency of orgasms). Our results are in accordance with the literature, observing an increase in the prevalence rates of dyspareunia and orgasmic difficulties in all types of hysterectomies performed. 31-33

In most developed countries, cancer is the second leading cause of death, preceded only by cardiovascular disease, and there is epidemiological evidence that this trend is emerging in developing countries. 1 The number of cancer deaths in the world is expected to increase 45% between 2007 and 2030 (from 7.9–11.5 million deaths), influenced, in part, by the increase in population and global aging. 34 The incidence of cancer tends to increase with age, probably due to the accumulation of risk factors for some specific cancers. 34 This susceptibility of the elderly can occur due to the duration of carcinogenesis, the vulnerability of the elderly tissues to environmental carcinogens, and other transformations that favor the development and growth of tumors. 35

Gynecological cancer survivors face several barriers with regard to identification and treatment of pelvic floor disorders, including communication issues between patient and health professional. 40 Hence, we highlight the importance of multidisciplinary programs to prevent and treat this group, providing information and strategies to improve vaginal symptoms and to improve sexual health.

Although most cervical cancer patients are not elderly, we need to be attentive to postoperative care for hysterectomized patients. This review indicates the importance of a thorough investigation involving the adverse effects of the treatment of hysterectomy. The quality of life should be increasingly valued, to the detriment of life in conditions limited or disabled. In this sense, the need for health assistance in the monitoring of these patients after the cancer diagnosis (both before and after surgery) is highlighted, to help with all these needs.

Regarding oncological patients, the biopsychosocial dimension must be considered. Thus, the literature indicates fear of disease recurrence, shame of urinary symptoms, reduced sexual function and vaginal motility, dyspareunia, and low sex drive. 41 Some of these subjects were addressed in the articles of our review, which reinforce the relevance of strategies to prevent harm to the psychological health of that group.

The limitations of this study were the inclusion of papers with non-comparable types of hysterectomies, with different follow-up times and measurement of different variables, and the inclusion of studies with a low level of evidence. Additional studies are needed to investigate the impact on the productivity of these patients' work, the causal relationships of the dysfunctions with the occupation and the carrying out of preventive interventions and conservative treatments aimed at this population.

## Conclusion

The urinary and sexual dysfunctions in hysterectomized women are frequent events. The main diseases reported in the studies were urinary incontinence, which had a prevalence variation between 7, in radical abdominal hysterectomy, and 47%, in laparoscopic radical hysterectomy (including stress and urge incontinence), and dyspareunia. The latter ranged from 5, in laparoscopic radical hysterectomy, to 19%, in laparoscopic nerve sparing radical hysterectomy.
