# Supplementary material for: The Effects of Hysterectomy on Urinary and Sexual Functions of Women with Cervical Cancer: A Systematic Review
Source: Rev Bras Ginecol Obstet. 2022 Sep 8;44(8):790–6. doi: 10.1055/s-0042-1748972 (PMC9948282; doi:10.1055/s-0042-1748972)
Supplement: Supplementary file 1 — Supplementary Material [file 10-1055-s-0042-1748972-s210373.pdf]

Chart 1 Prevalence of urinary and sexual symptoms in the articles included in the review, which assessed the effects of hysterectomy for malignant causes

No	Authors/Year	Evaluated symptoms and prevalence	Results	JBI
27	Elghamrawi et al. (2011)[26]	Urinary incontinence: ($p = 0.313$):<br>Radical surgery: 7%.<br>Radiotherapy: 16%.<br>Sexual dysfunction ($p = 0.145$):<br>Radical surgery: 20%.<br>Radiotherapy: 35%.	There was no difference in urinary complaints in patients in the group submitted to radiation ($p = 0.313$). The frequency of sexual dysfunction was compared between the two groups, with no statistical difference, but related to age ($p = 0.145$). The younger the patient's age, the greater the sexual complaint, regardless of the treatment modality. Sexual problems included dyspareunia due to vaginal shortening or stenosis, vulvar pain, and itching.	100%
36	Hoogendam et al. (2014)[35]	**LUTS:** ($p = 0.88$): 19%.<br>**Urinary tract infection:** 17%.<br>**Sexual dysfunctions (dyspareunia, decreased vaginal lubrication, reduced libido):** 9%.	LUTS (19%) and sexual dysfunction (9%) are present among the results of the study, such as long-term complications of assisted robotic laparoscopic radical hysterectomy, against early cervical cancer.	100%.
37	Plotti et al. (2018)[36]	**Urinary incontinence:**<br>28%	Regarding sexuality, the data indicated a good level of sexual pleasure involved with a slight worsening of sexual activity. Regarding urinary incontinence, this was reported in 28% of cases and appeared to be mild and rarely disabling.	100%
9	Selcuk et al. (2016)[9]	**Urinary incontinence:** ($p = 0.002$):<br>RAH: 29%.<br>ASH: 28%.<br>CONTROL: 11%.<br>**Urge incontinence:** ($p = 0.007$):<br>RAH: 16%.<br>ASH: 10%.<br>CONTROL: 3%<br>**Stress incontinence:** ($p = 0.575$):<br>RAH: 7%.<br>ASH: 8%.<br>CONTROL: 4%	Patients who underwent RAH had more irritating urinary symptoms than those in the control group ($p = 0.001$), there was also a significant difference when compared with SH ($p = 0.035$). The score for obstructive symptoms was also significantly higher in RAH ($p = 0.019$). Regarding the stress incontinence scores, there was no significant difference between the 3 comparison groups ($p = 0.890$ for RAH and $p = 0.375$ for the control group). Regarding sexual symptoms, women who underwent RAH had a significantly lower score than in the other groups ($p = 0.001$).	100%
38	Lalos et al. (2009)[37]	**Before treatment-**<br>Urinary incontinence: 26%.<br>Nocturia: 13%.<br>Dyspareunia: 6%.<br>**1 year after treatment:** ($p = 0.18$)<br>Urinary incontinence: 31%.<br>Nocturia: 18%.<br>Dyspareunia: 6%.	It was observed that there was no increase in the number of voluntary urination, urgency, and urinary incontinence episodes after 1 year of treatment ($p = 0.18$). Dyspareunia did not increase 1 year after treatment ($p = 0.05$).	100%
39	Noronha et al. (2012)[38]	**RH:**<br>Stress incontinence: 30%; ($p = 0.563$).<br>Urge incontinence: 45%; ($p = 0.549$).<br>Nocturia: 30%. ($p = 0.535$)<br>**RT:**<br>Stress incontinence: 45%; ($p = 0.563$).<br>Urge incontinence: 60%; ($p = 0.549$).<br>Nocturia: 40%. ($p = 0.535$).<br>**RT + QT:**<br>Stress incontinence: 30%; ($p = 0.563$).<br>Urge incontinence: 45%; ($p = 0.549$).<br>Nocturia: 60%. ($p = 0.535$).	The groups were similar in the incidence of LUTS ($p = 0.56$), urinary urgency ($p = 0.44$), urge incontinence ($p = 0.54$) and nocturia ($p = 0.53$). The QT / RT group had a higher urinary frequency ($p < 0.001$). The patients in the RH group were more sexually active ($p = 0.01$) and had less dyspareunia ($p = 0.021$). Vaginal length was shorter in the RT (5.5 ± 1.9 cm) and QT / RT (5.3 ± 1.5 cm) group than in the RH group (7.4 ± 1.1 cm) ($p < 0.001$). The muscular contraction of the pelvic floor was similar ($p = 0.302$).	100%
22	Pieterse et al. (2013)[21]	LRH: (p = NS):<br>**Before surgery-**<br>Urinary incontinence: 25%.	The results show that treatment against early cervical cancer results in an increase in subjective sexual and bladder	100%

 © 2022. Federação Brasileira de Ginecologia e Obstetrícia. All rights reserved.

Chart 1 (*Continued*)

No	Authors/Year	Evaluated symptoms and prevalence	Results	JBI
		Dyspareunia: 5%.<br>Difficulty having orgasm: 10%.<br>**12 months after surgery-**<br>Urinary incontinence: 35%.<br>Dyspareunia: 12%<br>Difficulty having orgasm: 19%.<br>**24 months after surgery-**<br>Urinary incontinence: 29%.<br>Dyspareunia: 16%.<br>Difficulty having orgasm: 14%.<br>LRH-NS: ($p = NS$).<br>**Before surgery-**<br>Urinary incontinence: 25%.<br>Dyspareunia: 7%.<br>Difficulty having orgasm: 9%.<br>**12 months after surgery-**<br>Urinary incontinence: 47%.<br>Dyspareunia: 19%.<br>Difficulty having orgasm: 19%.<br>**24 months after surgery-**<br>Urinary incontinence: 44%.<br>Dyspareunia: 17%.<br>Difficulty having orgasm: 13%.	symptoms, regardless of the surgical procedure used.	
16	Greer et al. (2010)[16]	TAH: ($p = NS$)<br>**Stress urinary incontinence (daily)** - 7% before surgery and 11% after surgery.<br>**Urge incontinence (daily)** - 7% before surgery and 5% after surgery.<br>Supracervical hysterectomy: ($p = NS$).<br>**Stress urinary incontinence (daily)** - 11% before surgery and 11% after surgery.<br>**Urge incontinence (daily)** - 7% before surgery and 5% after surgery.	There was no difference in prevalence between pre and postoperative in the two techniques.	62.5%

Abbreviations: ASH, abdominal subtotal hysterectomy; LRH – NS, laparoscopic radical hysterectomy – nerve sparing; LRH,l radical hysterectomy; LUTS, lower urinary tract symptoms; QT, chemotherapy; RAH, radical abdominal hysterectomy; RH, radical hysterectomy; RT, radiotherapy; TAH, total abdominal hysterectomy.

 © 2022. Federação Brasileira de Ginecologia e Obstetrícia. All rights reserved.

Chart 2 Characterization of the articles included in the sample, which evaluated the effects of hysterectomy for malignant causes

N°	Authors/year	Country	Objective	Study design	Sample	Average Age	Data collection instrument and / or quality of life	Measuring instruments	Follow-up period (PRE AND POSTSURGERY)
**27**	Elghamrawi et al. (2011)[26]	Egypt	To report the quality of life and late effects of radiotherapy or surgery among women treated for cervical cancer and previously free of disease, for 5 years or more.	Retrospective cohort	TAH: ($n=41$) RT: ($n=57$)	98 women, with an average age of 50.8 years, diagnosed and treated against cervical cancer (stage IA, IIA, and IIB), without metastasis and free of the disease for 5 years or more, on the date of data collection.	WHO scoring system	–	5 years after treatment.
**36**	Hoogendam et al. (2014)[35]	Netherlands	Report the oncological outcome and long-term complications of robotic laparoscopic radical hysterectomy assisted in the early stage of cervical cancer.	Retrospective cohort	–	100 patients with primary cervical cancer, treated at a tertiary referral center. Average age: 41 years.	CTCAE scale, version 4.3.	Anamnesis and complete physical examination.	Follow-up of 3 months (in the first year), 4 months (in the second year), and 6 months in the past 3 years.
**37**	Plotti et al. (2018)[36]	Italy	To investigate long-term quality of life and long-term urinary and sexual function in cervical cancer survivors previously treated with radical abdominal hysterectomy.	Retrospective cohort	–	90 patients diagnosed with locally advanced cervical cancer (stage IB2 - IVA), complete response to treatment 36 months later, sexually active, without chronic diseases, without a history of another cancer, without pelvic inflammatory disease and without the use of antidepressants. Average age: 55.6 years.	QLQ-CX24, QLQ-C30 questionnaire e Incontinence Impact Questionnaire	–	49 months after the end of treatment.
**09**	Selcuk et al. (2016)[9]	Turkey	To assess the impact of simple abdominal and radical abdominal hysterectomy on all aspects of pelvic floor dysfunction.	Retrospective cohort	**TAH:** 41 patients undergoing radical abdominal hysterectomy to treat cervical cancer. **SAH:** 58 patients undergoing simple abdominal	142 women. **Average age**: TAH: 52.49 years. SAH: 50.34 years. NO SURGERIES: 49.21 years.	Urogenital distress Inventory (UDI-6); Incontinence impact questionnaire (IIQ-7); Pelvic floor and incontinence sexual impact questionnaire (PISQ-12).	–	It does not appear in the text.

 © 2022. Federação Brasileira de Ginecologia e Obstetrícia. All rights reserved.

Chart 2 (*Continued*)

N°	Authors/year	Country	Objective	Study design	Sample	Average Age	Data collection instrument and / or quality of life	Measuring instruments	Follow-up period (PRE AND POSTSURGERY)
					hysterectomy to treat benign conditions.<br>**NO SURGERIES:** 43 women who have not undergone any surgery.				
**38**	Lalos et al. (2009)[37]	Sweden	Search for information on the occurrence of urinary, climacteric, and sexual symptoms in women with cervical cancer.	Prospective cohort	–	39 women with cervical cancer, mean age 43 years.	Questionnaires standardized by the research institution		Before treatment.<br>1 year after treatment.
**39**	Noronha et al. (2012)[38]	Brazil	Describe the impact of surgery, radiotherapy and chemoradiation on pelvic floor functions in patients with cervical cancer.	Prospective cohort	**TAH:** 20 Women undergoing radical hysterectomy.<br>**RT**: 20 Women undergoing radiation therapy<br>**QT + RT**: 20 Women undergoing chemoradiation	60 patients.<br>**Average age:**<br>TAH: 50.3 years<br>RT: 52.9 years<br>QT + RT: 51.5 years	Questionnaires standardized by the research institution	–	It does not appear in the text.
**22**	Pieterse et al. (2013)[21]	Netherlands	To evaluate self-reported morbidity due to various types of treatment in patients with cervical cancer.	Prospective cohort	**LRH NS:** 123 undergoing radical laparoscopic hysterectomy with nerve preservation.<br>**LRH**: 106 undergoing conventional laparoscopic radical hysterectomies	229 patients.<br>Average age:<br>LRH NS: 45 years<br>LRH: 43.57 years	Dutch Gynecologic Leiden Questionnaire	–	Before treatment.<br>1 year after treatment.<br>2 years after treatment.
**16**	Greer et al. (2010)[16]	England	Second to an original study, lower urinary tract symptoms, POP, sexual function and quality of life, 9 years after total abdominal hysterectomy and total supracervical hysterectomy.	Cross-sectional	**TAH:** 27 who underwent total abdominal hysterectomy.<br>**SUPRACERVICAL HYSTERECTOMY**: 27 who underwent total supracervical hysterectomy.	54 women.<br>**Average age:**<br>TAH: 40.8 years<br>SUPRACERVICAL HYSTERECTOMY: 41.8 years	Questionnaires used in the original study	–	9 years after hysterectomy

Abbreviations: CTCAE, common terminology criteria for adverse event; QT, chemotherapy; RLH, radical laparoscopic hysterectomy; RLH-NS, radical laparoscopic hysterectomy- nerve sparing; RT, radiotherapy; SAH, simple abdominal hysterectomy; TAH, total abdominal hysterectomy.

 © 2022. Federação Brasileira de Ginecologia e Obstetrícia. All rights reserved.
